# Impaired Autophagy and Defective Mitochondrial Function: Converging Paths on the Road to Motor Neuron Degeneration

**DOI:** 10.3389/fncel.2016.00044

**Published:** 2016-03-03

**Authors:** Brittany M. Edens, Nimrod Miller, Yong-Chao Ma

**Affiliations:** ^1^Departments of Pediatrics, Neurology, and Physiology, Northwestern University Feinberg School of MedicineChicago, IL, USA; ^2^Lurie Children’s Hospital of ChicagoChicago, IL, USA

**Keywords:** autophagy, mitochondria, calcium homeostasis, protein aggregation, ATP biogenesis, oxidative stress, ALS, motor neuron disease

## Abstract

Selective motor neuron degeneration is a hallmark of amyotrophic lateral sclerosis (ALS). Around 10% of all cases present as familial ALS (FALS), while sporadic ALS (SALS) accounts for the remaining 90%. Diverse genetic mutations leading to FALS have been identified, but the underlying causes of SALS remain largely unknown. Despite the heterogeneous and incompletely understood etiology, different types of ALS exhibit overlapping pathology and common phenotypes, including protein aggregation and mitochondrial deficiencies. Here, we review the current understanding of mechanisms leading to motor neuron degeneration in ALS as they pertain to disrupted cellular clearance pathways, ATP biogenesis, calcium buffering and mitochondrial dynamics. Through focusing on impaired autophagic and mitochondrial functions, we highlight how the convergence of diverse cellular processes and pathways contributes to common pathology in motor neuron degeneration.

## Introduction

Amyotrophic lateral sclerosis (ALS) is an adult-onset neurodegenerative disease characterized by loss of large motor neurons in the brain and spinal cord, resulting in progressive voluntary muscle wasting and respiratory failure. Patient death typically ensues 3–5 years following symptom onset (Volonte et al., 2015). With a prevalence of four-to-six per 100,000 people affected worldwide, ALS is one of the most common neurodegenerative disorders (Tan et al., 2007). Its origins can be either familial or sporadic, of which familial forms account for a mere 10% whereas the remaining 90% are sporadic (Rotunno and Bosco, 2013). Although ALS was first described by Charcot as early as 1869 (Jay, 2000), it wasn’t until more than a century later that the first casual mutation was identified in copper zinc superoxide dismutase 1 (*SOD1*; Rosen, 1993). Since then, a multitude of genes associated with ALS pathogenesis have been identified. Nonetheless, mechanisms underlying motor neuron-specific vulnerability in ALS remain largely unknown. At present the disease defies all treatment. Riluzole is the only FDA-approved drug for treating ALS, and it may prolong patient lifespan by mere months (Rowland and Shneider, 2001).

Despite the heterogeneous and multigenic nature of ALS, overlapping pathology and common phenotypes are observed in different forms of the disease. Protein aggregates found in ALS patients suggest that cellular clearance mechanisms, such as the autophagy-lysosome pathway, may be impaired in this disease (Blokhuis et al., 2013). Moreover, increased oxidative stress and compromised mitochondrial function are observed in ALS disease condition (Wang and Michaelis, 2010). Intriguingly, oxidative stress is a potent regulator of autophagy (Scherz-Shouval et al., 2007; Huang et al., 2011; Scherz-Shouval and Elazar, 2011), suggesting the potential for functional interactions between the lysosomal and mitochondrial pathways.

Herein, we will consider in depth the dysregulation of autophagy and mitochondrial pathways, as well as their interactions in the context of ALS pathogenesis. We will put a special emphasis on mitophagy, as it directly connects cellular clearance mechanisms with mitochondrial function. Then, we will review the wealth of information regarding mitochondrial dysfunction in ALS, with particular interest in data derived from various mouse models. Lastly, we will discuss the role of oxidative stress as a critical regulator linking these discrete processes, and consider the therapeutic implications for ALS.

## Mechanisms of Cellular Clearance in Physiological and Pathological Conditions

### Autophagy

Autophagy is a catabolic process by which cells degrade and recycle cellular constituents through lysosomes to balance sources of energy and building blocks in order to maintain cellular homeostasis and function (Ryter et al., 2013; Yang et al., 2013). The core autophagy machinery consists largely of autophagy-related (ATG) genes, of which ATG1–10, ATG12–16, and ATG18 are all required (Klionsky et al., 2003; Mizushima et al., 2011). Beginning with induction, autophagy is initiated by intracellular or extracellular stimuli such as nutrient deprivation or stress. The most upstream player in the induction process is ATG1, whose mammalian homologs are unc51-like kinase 1 and 2 (ULK1 and 2; Mizushima, 2010). ULK1 interacts with Atg13, FIP200 (focal adhesion kinase family interacting protein of 200 kD), and ATG101 to form an autophagy-initiating complex (Hara et al., 2008; Hosokawa et al., 2009).

A major regulatory event in autophagy induction is exerted by the initiation complex’s interactions with the nutrient-sensing mTOR kinase, and the energy-sensing AMP-activated protein kinase (AMPK). AMPK, activated by a drop in cellular energy, phosphorylates ULK1 on Serine 317 and Serine 777 (Hawley et al., 1996; Egan et al., 2011). These phosphorylation events in turn activate ULK1, which initiates autophagy (Egan et al., 2011). Conversely, the presentation of nutrients activates mTORC1 (through amino acid binding), which phosphorylates ULK1 on Serine 757, leading to the inhibition of autophagy (Kim et al., 2011). Therefore, autophagy initiation is kept in check by both nutrient- and energy-sensing mechanisms.

Following induction, the autophagosome forms and sequesters substrates for degradation. Autophagosome formation is controlled by ATG5 and ATG12/ATG16, which conjugate to recruit LC3 (microtubule-associated protein 1A/1B-light chain 3), a well-established autophagosomal marker (Romanov et al., 2012). In addition, a protein complex consisting of Beclin1, ATG14L and VPS34, a class III phosphoinositide 3-kinase (PI3K), has also been shown to play a critical role in autophagosome formation by serving as a scaffold to recruit autophagy targets to the autophagosome lumen (Volinia et al., 1995; Obara and Ohsumi, 2011). The active ULK1 initiation complex positively regulates autophagy at this level by phosphorylating Beclin-1 on Serine14 to promote the activity of VPS34 (Russell et al., 2013).

**Figure 1:**
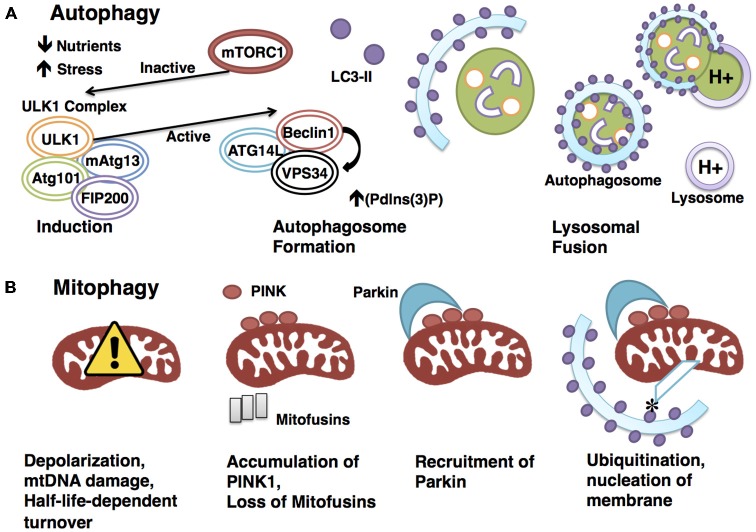
**Degradative pathways are tightly regulated. (A)** Autophagy is a highly regulated catabolic process. Induction is triggered by physiologically relevant events, such as a drop in nutrients or a rise in stress. mTORC1 represses induction through interaction with the ULK1 initiation complex until an appropriate signal is sensed, at which point mTORC1 rapidly dissociates to allow the activation of the ULK1 complex. The activated ULK1 complex goes on to phosphorylate Beclin1, which in turn positively regulates the activity of VPS34. This results in increased (Pdlns(3)P), a requirement for autophagosome formation. The LC3-II-studded autophagosome membrane then envelops cytoplasm before docking to and fusing with the acid hydrolase-containing lysosome, a process that will enable degradation of the sequestered cytoplasmic materials. **(B)** Mitophagy requires the canonical autophagy pathway constituents with the addition of mitochondrial-specific adaptors to confer specificity. Targeting of mitochondria for degradation in the PINK1-Parkin-dependenr pathway illustrated here begins with depolarization. Polarization-dependent cleavage of PINK1 is halted, allowing the kinase to accumulate on the outer membrane and recruit the E3 ubiquitin ligase Parkin. PINK1 phosphorylates Parkin, and Parkin ubiquitinates mitochondrial outer-membrane proteins, such as mitofusins. Ubiquitination allows for the docking of adaptor proteins such as optineurin, which recruits LC3 to the targeted mitochondrion to form the autophagosome.

The process of autophagy is completed when the mature autophagosome docks to and fuses with the lysosome, where its cargo are degraded to release energy and cellular building blocks (Mizushima, 2007; Figure 1). This pathway and many of its key molecular constituents are highly conserved throughout evolution, indicating the vital importance of autophagic functions. The most pertinent functions of autophagy include clearance, which eliminates damaged organelles and long-lived proteins that would otherwise compromise cellular health; and catabolism, which releases energy and building blocks to support the growth and activity of the cell (Singh and Cuervo, 2011).

### Cellular Clearance and Neurodegeneration

Though the process of autophagy is ubiquitous throughout a diversity of cells and species, its importance in neurons has become increasingly apparent (Wang and Hiesinger, 2012; Yang et al., 2013). Degradative pathways are essential to maintain balances of cellular energy and stress to promote homeostasis (Ryter et al., 2013); demands on homeostatic regulation are particularly high in neuronal tissue (Chen et al., 2012; Le Masson et al., 2014). As a hallmark of numerous neurodegenerative diseases, the formation of neuronal aggregates highlights the importance of protein clearance in maintaining neuronal health (Polymenidou and Cleveland, 2012; Lim and Yue, 2015). Common to all patients as well as models of ALS is the presentation of pronounced protein inclusions, despite the complex genetic state and broad spectrum of associated mutations involved in the disease. Proteins associated with these aggregates include FUS, TDP-43, UBQLN2 and SOD1, among others (Deng et al., 2011b; Blokhuis et al., 2013). These aggregates may exhibit toxic properties in addition to afflicting the cell by maintaining long-lived proteins (Sau et al., 2007; Johnson et al., 2008; Xu et al., 2013; Wu et al., 2014), as demonstrated in *SOD1* mutant mice, where aggregation was shown to drive toxicity in motor neurons. The potency of this toxicity was exacerbated by proteasomal dysregulation (Bruijn et al., 1998; Kitamura et al., 2014).

Decreased efficiency of autophagy, as suggested by the presentation of aggregates, has long been correlated with neurodegeneration. However, a causative relationship between defective autophagy and neurodegeneration was not established until the independent findings that neuronal dysfunction and pathology follow the loss of either *Atg5* or *Atg8* in mouse (Hara et al., 2006; Komatsu et al., 2006). In both studies, knockout was restricted to the nervous system. Both *Atg5*- and *Atg8*-deficient mice presented with similar phenotypes: profound motor impairment was noted by 3 and 4 weeks of age, manifesting as impaired coordination, poor balance and reduced grip strength. Moreover, both models exhibited the limb-clasping reflex upon suspension, an aberrant behavior noted previously in rodent models of neurodegeneration. Upon histological analysis, brains from both models revealed a high degree of cellular degeneration, accompanied by ubiquitin-positive inclusions and the accumulation of ubiquitinated proteins. These findings strongly suggest that the reduction of basal protein turnover is sufficient to render cells vulnerable to degeneration.

The link between inefficient protein clearance and neurodegeneration is further illustrated by the nature of the mutations resulting in pathological states. Mutations identified in *UBQLN2* have been linked to ALS/FTD (Deng et al., 2011b), as well as a more heterogeneous spectrum of neurodegenerative diseases more recently (Fahed et al., 2014). Aggregation of ubiquilin2 protein is found not only in individuals afflicted with heritable *UBQLN2* mutations, but also in unrelated sporadic ALS patients and mouse models, suggesting a general role for *UBQLN2* in ALS pathogenesis (Deng et al., 2011a). Ubiquilins are ubiquitin-like proteins implicated in regulating autophagy, as well as the ubiquitin proteasome system (UPS; Zhang et al., 2014). They have been shown to colocalize with autophagosomes and associate with LC3 (N’Diaye et al., 2009); furthermore, reduction of ubiquilin levels correlates with a decrease in autophagosome number, suggestive of a critical role in autophagosome formation (Rothenberg et al., 2010). Precisely how and to what extent ubiquilins participate in autophagy, and what implications this has for ALS pathology are exciting questions requiring further attention.

*p62/SQSTM1* is another ALS-associated gene involved in degradative pathways (Teyssou et al., 2013). Like *UBQLN2*, it is associated with both the UPS and autophagy. In the ubiquitin pathway, p62 delivers polyubiquitinated substrates to the proteasome (Seibenhener et al., 2004). In autophagy, p62 interacts directly with LC3 and ATG8 to confer specificity of targeting by selectively acquiring a subset of polyubiquitinated proteins (Pankiv et al., 2007). Just as ubiquilin2-positive inclusions have become a general observation in both familial and sporadic ALS, so have p62-positive inclusions (Mizuno et al., 2006; Deng et al., 2011a), thus signifying the requirement for protein clearance pathways in the maintenance of neuronal health (Fecto and Siddique, 2012).

In recent years mutations in chromosome 9 open reading frame 72 (*C9orf72*) have emerged as the most common cause of both sporadic and familial ALS (DeJesus-Hernandez et al., 2011; Renton et al., 2011). Though much about this peculiar gene awaits characterization, it is proposed to function as a small GTPase Rab GEF (Guanine Nucleotide Exchange Factor), and has been recently implicated in endosomal trafficking (Zhang et al., 2012; Farg et al., 2014). Furthermore, *C9orf72* colocalizes with ubiquilin2 and LC3-positive vesicles, as well as autophagy-related small GTPase Rabs, suggesting a potential role in autophagy. Further studies of *C9orf72* will be necessary to elucidate its function in this process, as well as suggest a mechanism of pathogenesis in ALS.

### Mitophagy

Mitophagy refers to the selective process whereby mitochondria are targeted and degraded by the autophagy machinery, primarily for the purpose of eliminating defective organelles. Though the key components and sequence of events are largely conserved between mitophagy and autophagy, mitophagy requires additional mitochondrial-specific adaptors to ensure specificity of targeting (Kim et al., 2007).

The best-studied mitophagy pathway is mediated by PTEN-induced putative protein kinase 1 (PINK1) and Parkin. Mutations in *PINK1* and *PARK2*, the gene encoding Parkin, result in pronounced mitochondrial dysfunction, leading to degeneration of muscle and neurons (Poole et al., 2008; Jin and Youle, 2012; Figure 1). The finding that overexpression of Parkin is sufficient to mitigate *PINK1* mutant phenotypes suggests interaction in the same pathway, with PINK1 upstream of Parkin (Greene et al., 2003; Pesah et al., 2004; Clark et al., 2006). PINK1 is a serine/threonine kinase that associates with mitochondria via a conserved targeting sequence. In healthy mitochondria, PINK1 is imported from the outer to the inner membrane where it is cleaved in a membrane polarization-dependent manner (Deas et al., 2011). When the mitochondrial membrane potential is compromised, PINK1 can no longer be cleaved, thus it accumulates on the outer membrane (Narendra et al., 2010), serving as a signal for recruitment of the E3 ubiquitin ligase Parkin (Vives-Bauza et al., 2010; Shiba-Fukushima et al., 2012). PINK then phosphorylates Parkin and ubiquitin, which is required for Parkin’s E3 ligase activity (Koyano et al., 2014). The mechanism underlying phosphorylated ubiquitin activation of Parkin has recently been elucidated (Wauer et al., 2015), following the crystallization of the complex comprising Parkin and phosphorylated ubiquitin. Structural analysis revealed that the binding of phosphorylated ubiquitin to Parkin results in a conformational change within the RING domain, which ultimately results in the activation of Parkin. Importantly, Parkin’s ubiquitin binding pocket is commonly mutated in autosomal-recessive Parkinson’s, indicating the vital nature of this interaction in promoting mitophagy.

The downstream events that link the targeting of depolarized mitochondria by PINK1-Parkin to the canonical autophagy core machinery are not yet entirely clear. Direct interaction of Parkin with Beclin1 represents one possible explanation (Khandelwal et al., 2011; Lonskaya et al., 2013). Additionally, Parkin has been demonstrated to recruit the Beclin1 regulator AMBRA1 (Activating molecular in BECN1-regulated autophagy protein 1) to the outer membrane of depolarized mitochondria, an event that could also activate the Beclin1 complex and the core autophagy machinery (Van Humbeeck et al., 2011). Interestingly a role for PINK1 was recently discovered in the recruitment of the autophagy receptors optineurin and NDP52 to depolarized mitochondria. This was shown to activate mitophagy in a Parkin-independent manner, whereby optineurin and NDP52 recruit ULK1 and other autophagy factors (Lazarou et al., 2015).

A number of PINK1-Parkin-independent mechanisms of mitophagy have been described. A reduction in iron, for example, has been shown to promote a distinct pathway that does not rely on PINK1-Parkin. This novel pathway involves the transition from oxidative phosphorylation (OXPHOS) to glycolysis and induction of mitophagy without compromising membrane polarization. Importantly, PINK1 stabilization is not required for this process; iron chelation-induced mitophagy is efficiently activated in fibroblasts deficient in Parkin (Allen et al., 2013). Another PINK1-Parkin-independent pathway is suggested by findings on AMBRA1: Parkin is not required for a distinct form of ABMRA1-mediated mitophagy. Though AMBRA1 is known to interact with Parkin, which is thought to contribute to canonical PINK1-Parkin-mediated mitophagy, it has been shown that mitochondrial-targeted AMBRA1 is sufficient to induce mitophagy in a Parkin-free system, therefore representing a novel mitophagic pathway (Strappazzon et al., 2015). Finally, a mechanism of PINK1-Parkin-independent hypoxia-induced mitophagy has been identified. The mitochondrial outer-membrane protein FUNDC1, FUN14 domain-containing protein 1, is a mitophagy receptor requiring LC3 interaction. Either knockdown of FUNDC1 or mutation of its LC3-binidng domain significantly hinders hypoxia-induced mitophagy (Liu et al., 2012). Given our growing knowledge of the roles mitochondrial quality control plays in neuronal maintenance, further elucidation of mitophagic pathways will be necessary to advance our understanding and treatment of neurodegenerative disease.

The strongest evidence supporting the contribution of impaired mitophagy to ALS pathogenesis lies in the associated mutations. The involvement of optineurin, encoded by the *OPTN* gene, in ALS pathogenesis was identified years after the gene had been implicated in primary open-angle glaucoma (Maruyama et al., 2010). Optineurin is involved in a number of cellular processes including Golgi maintenance and membrane trafficking, but its function as an autophagy receptor is presumably the most relevant to ALS pathogenesis (Turturro et al., 2014). Recently, optineurin was shown to play a significant role in PINK1-Parkin-mediated mitophagy. Damaged mitochondria are initially targeted by the E3 ubiquitin ligase Parkin, which ubiquitinates outer membrane proteins such as mitofusins, the outer membrane-embedded GTPases responsible for mediating mitochondrial fusion (Poole et al., 2010). Optineurin then binds to the ubiquitinated mitochondrial outer membrane proteins with its ubiquitin-binding domain. Hereafter, optineurin induces nucleation of the autophagosome by recruitment of LC3. ALS-causing mutations in *OPTN* disable this process, implicating inefficient mitochondrial clearance in ALS (Wong and Holzbaur, 2014). It is intriguing that, much like ubiquilin2 and p62, optineurin has been localized to inclusions in both familial and sporadic ALS (Blokhuis et al., 2013), suggesting a broader role for optineurin, and mitophagy, in ALS pathogenesis.

Valosin containing protein (VCP) mutations have been linked with ALS and other degenerative diseases, including dementia (Johnson et al., 2010; Koppers et al., 2012). VCP is a type II ATPase involved in a broad spectrum of biological processes ranging from proteasomal degradation and mitochondrial quality control via mitophagy, to endoplasmic reticulum (ER)-associated degradation (Dai and Li, 2001; Rabinovich et al., 2002; Wojcik et al., 2006; Ju et al., 2009). VCP lies downstream of the E3 ubiquitin ligase Parkin and is recruited to the outer membrane of damaged mitochondria. Degeneration-associated mutant VCP or loss of VCP results in the failure of PINK1-Parkin-mediated mitochondrial clearance. Moreover, VCP has been demonstrated to regulate the proteasomal degradation of mitofusins (Kim et al., 2013). Altogether, the role of optineurin and VCP in mitochondrial quality control supports dysregulation of mitophagy as a critical mechanism in ALS pathogenesis.

Very recently, two independent studies identified a definitive link between TBK1, TANK Biding Kinase 1, and ALS, citing a loss-of-function mechanism-of-action (Cirulli et al., 2015; Freischmidt et al., 2015). Substrates of TBK1 include optineurin as well as p62, thus strengthening the connection between mitophagic function and motor neuron degeneration in ALS. Indeed, mutations resulting in disruption of the C-terminal coiled-coiled optineurin-interacting domain of TBK1 were linked with pathogenesis, suggesting a vital role for the kinase in the regulation of mitophagy. Accordingly, a recent study has elucidated the mechanism by which TBK1 acts in mitophagy (Heo et al., 2015). PINK1-Parkin-dependent phosphorylation of TBK1 activates the kinase to recruit autophagy receptors optineurin and NDP52 to the depolarized mitochondrion. TBK1 phosphorylates these receptors, and in turn, optineurin binding to polyubiquitin chains on the mitochondrial outer membrane enhances TBK1 activation, thus amplifying the mitophagic signal. Importantly, TBK1-dependent phosphorylation of optineurin increases its affinity for polyubiquitin binding, and this step is required for efficient mitophagy. Altogether, these findings put forth a novel framework within which TBK1 plays an integral role in amplifying the mitophagic signal via enhanced recruitment and activation of autophagy receptors on depolarized mitochondria, linking dampened mitophagy with ALS pathogenesis.

## Mitochondrial Function: Impairments Implicated in ALS

### ATP Generation

ATP is produced in mitochondria through OXPHOS of glucose via the electron transport chain (ETC). In this process, electrons pass along a sequence of protein complexes (I-IV) located in the inner mitochondrial membrane (Milstein and Swaiman, 1968). A flow of protons is pumped from the matrix into the inter-membrane space, generating an electrochemical gradient. When protons flow from high to low concentration through ATP synthase on the inner mitochondrial membrane, ADP is converted to ATP, storing high-energy in a phosphate bond for later use. Linking ATP biogenesis with ALS, a computational modeling endeavor suggested a framework within which mitochondrial dysfunction driving reduced ATP availability underlies motor neuron susceptibility to degeneration. According to this model, ATP reduction drives hyperexcitability through mechanisms such as Na^+^/K^+^ dyshomeostasis, and intracellular calcium hikes (Le Masson et al., 2014).

Reduced ETC activity has likewise been described in both patients and models of ALS, and may significantly contribute to pathogenesis. Compared to healthy controls, ALS-derived fibroblasts show altered mitochondrial bioenergetics: membrane potential is significantly reduced in correlation with age of onset, and an overall decrease in mitochondrial content is noted when compared to controls (Kirk et al., 2014). In line with these findings, a mutant SOD1-mediated switch from OXPHOS to glycolysis has been reported, indicative of inefficient ATP biogenesis in ALS (Allen et al., 2014). Direct genetic evidence of this comes from the recent identification of mutations in *CHCHD10* causing ALS and frontotemporal dementia (Bannwarth et al., 2014; Chaussenot et al., 2014). Though the functional repertoire of *CHCHD10* awaits further elucidation, it is predicted to play a significant role in energy production through OXPHOS (Bannwarth et al., 2014).

The wealth of data leading to our current understanding of mitochondrial dysfunction in ALS originates from reports on the *SOD1* class of mutations (Bendotti and Carri, 2004). These mutations are the most widely studied in ALS, as *SOD1* was the first identified FALS-linked gene (Rosen et al., 1993). Wild-type SOD1 is found predominantly in the cytoplasm where it neutralizes the damaging effects of reactive oxygen species (ROS; Fukai and Ushio-Fukai, 2011). A small amount of SOD1 is localized to the mitochondrial inter-membrane space (Jaarsma et al., 2001) where it is suggested to play a protective role in motor neurons (Waterman-Storer et al., 1997). ALS-associated mutations lead to increased localization of misfolded SOD1 protein to the mitochondrial inter-membrane space (Liu et al., 2004; Deng et al., 2006), and mutant SOD1-mitochondrial interactions lead to the alteration of mitochondrial redox potential (Ferri et al., 2006). Accumulation of SOD1 mutant aggregates has been shown to lead to dysfunctional mitochondria with decreased ATP production, calcium buffering and motility defects (Cozzolino and Carri, 2012). Furthermore, OXPHOS activity is impaired in both ALS patients and *SOD1* mutant transgenic mice (Bacman et al., 2006). Reduced levels of respiratory chain activity in complexes I-IV were observed in spinal cord tissue collected from ALS patients (Wiedemann et al., 2002). In the *SOD1^G93A^* mouse model, a pre-symptomatic decrease in Complex I function was noted, while Complex IV was found to be impaired following ALS-like symptom onset (Jung et al., 2002).

### Calcium Buffering

In motor neurons, mitochondria must meet not only high energy demands but also considerable calcium buffering requirements. Healthy mitochondria have enormous calcium buffering capacity and are able to effectively travel from areas of low calcium to areas of high calcium to restore normal levels (Wang and Schwarz, 2009). When mitochondria sense an increase in calcium they become stationary by arresting mitochondrial movement (Rintoul et al., 2003; Szabadkai et al., 2006). As one of the most eminent second messengers in neuronal cells, calcium is required by neurons for neurotransmitter release, modulation of synaptic efficiency, and signal transduction. Unregulated accumulation of calcium proves toxic to cells, however. In neurons, when excitatory glutamate receptors become excessively stimulated, high levels of calcium flow into the cell, leading to cell damage and death (Carriedo et al., 2000; Corona and Tapia, 2007). How calcium mediates this effect is not entirely understood, but its role in enzymatic and signaling cascade activation is one possible explanation.

Dysregulation of calcium homeostasis has been extensively reported in ALS, particularly as it pertains to *SOD1* mutation (Jaiswal and Keller, 2009). There have been reports of chronic calcium overload in mitochondria at nerve terminals (Siklós et al., 1996), and *SOD1* mutant mouse nerve terminals were shown to be depolarized as a result (Nguyen et al., 2009). Additionally, decreases in calcium loading capacity occur in the spinal cord and brain of mutant *SOD1* mice (Damiano et al., 2006). Calcium dysregulation was also linked to SOD1 mutant protein aggregation and impaired mitochondrial movement (Tradewell et al., 2011). Therefore, excitotoxicity following calcium dysregulation is posited to be a prominent mechanism in ALS pathogenesis (Van Den Bosch et al., 2006). While widely supported, this view faces opposition from a study identifying early (presymptomatic), but not late (endstage) hyperexcitability in the context of altered calcium handling (Fuchs et al., 2013). Such findings therefore call into question the role of hyperexcitability in motor neuron cell death.

Motor neurons exhibit a low calcium buffering capacity, largely the result of sparse calcium buffering protein expression (Celio, 1990; Ince et al., 1993; Palecek et al., 1999). Moreover, calcium handling and buffering capacity are impaired in models of ALS (Jaiswal et al., 2009; von Lewinski et al., 2008). Whereas proteins such as parvalbumin and calbindin are abundant in degeneration-resistant neuronal subtypes, conferring more stable calcium homeostasis (Vanselow and Keller, 2000), expression is comparatively limited in the motor neurons affected in ALS (Elliott and Snider, 1995). What is more, the intracellular free calcium increase noted in *SOD1^G93A^* transgenic mice at both late presymptomatic and symptomatic stages was correlated with decreased expression of calcium-buffering proteins such as SERCA1, SERCA2, and parvalbumin (Chin et al., 2014). Additionally, calcium-permeable α-Amino-3-hydroxy-5-methyl-4-isoxazolepropionic acid receptor (AMPAR) expression, especially those lacking the GluR2 subunit, is particularly high in motor neurons, permitting heightened calcium influx (Lips and Keller, 1998; Van Damme et al., 2002). This may contribute to the susceptibility of cultured motor neurons to AMPAR-mediated excitotoxicity upon overstimulation (Carriedo et al., 1996, 2000; Corona and Tapia, 2007). Accordingly, the anti-excitotoxic drug, Riluzole, is at present the only agent approved for the treatment of ALS, which functions by blocking AMPA receptors, thus inhibiting glutamate release and regulating calcium levels in the cytosol (Bellingham, 2011). When tested in the *SOD1* mutant mouse model, Riluzole showed an increase in survival (Gurney et al., 1996), but in patients it has been shown to expand life by only several months on average (Bensimon et al., 1994; Lacomblez et al., 1996). It is worth noting that, despite the well-established role of altered calcium handling in ALS, enhanced buffering as achieved by elimination of cyclophilin D was insufficient to extend survival in *SOD1* mouse models, in spite of improved motor neuron survival and reduced SOD1 aggregation (Parone et al., 2013).

### Mitochondrial Transport

**Figure 2:**
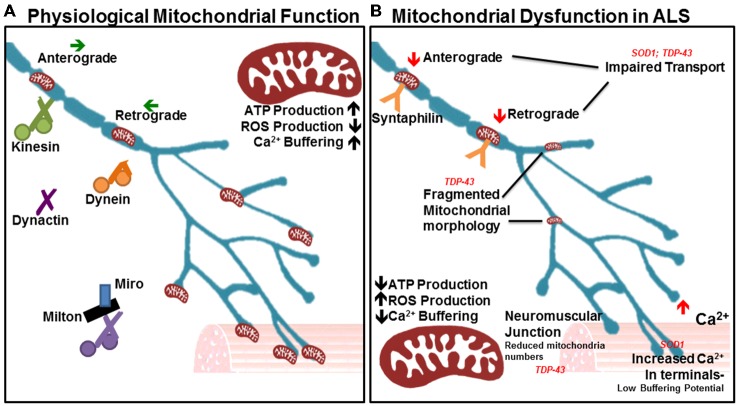
**Many aspects of mitochondrial function are impaired in amyotrophic lateral sclerosis (ALS). (A)** In physiological states, mitochondria show a high capacity for ATP production and Ca^2+^ buffering, with low reactive oxygen species (ROS) production. The orderly anterograde and retrograde trafficking of mitochondria along axons by motor proteins ensures that energy and calcium buffering are appropriately mobilized given the local needs throughout the cell and neurites. Milton/Miro and syntaphilin dock mitochondria in accordance with localized energy or buffering needs. Their dense distribution is noted in the neuromuscular junction, as well as other areas of high Ca^2+^ and metabolic demand. **(B)** In ALS, mitochondrial ATP production and Ca^2+^ buffering capacity are reduced, while ROS production is elevated. Accordingly, Ca^2+^ levels are heightened in motor nerve terminals. Impairments in axonal transport of mitochondria are noted, as is a depletion of the mitochondrial population at the neuromuscular junction. Mitochondrial morphology is also irregular in diseased motor neurons, indicating poor health and inefficient turnover.

Motor neurons innervate muscle fibers great distances away from the soma, which requires mitochondria to be continually transported along the axon to areas of high energy and calcium buffering demand. Defects in mitochondrial transport render neurons especially vulnerable to degeneration (De Vos et al., 2008; Wang and Schwarz, 2009). Mitochondria utilize many different motor proteins to move in a saltatory manner, which enables their recruitment to areas of low ATP where they remain docked due to high levels of ADP that halt movement (Mironov, 2007). Generally, about one-third of mitochondria are in motion while two-thirds are stationary (Sheng, 2014). Mitochondria travel along microtubules either away from the soma (anterograde) or towards the soma (retrograde). Kinesins are generally responsible for anterograde travel whereas dynein facilitates retrograde transport (Forman et al., 1983). Other key players in mitochondrial transport include dynactin (Waterman-Storer et al., 1997), syntaphilin (Kang et al., 2008), and the Milton-Miro complex (Stowers et al., 2002; Figure 2). Intriguingly, neuronal-restricted loss of Miro culminates in brain stem and spinal cord atrophy resembling motor neuron disease, suggesting a requirement for mitochondrial mobility in motor neuron health and maintenance (Nguyen et al., 2014).

Mitochondria in the ALS-linked *SOD1* mutant mouse model experience axonal transport deficits before ALS symptoms arise, resulting in a deficiency of axonal mitochondria (De Vos et al., 2007). Multiple studies have shown impairment in anterograde and retrograde transport (Bilsland et al., 2010; Magrané et al., 2012; Marinkovic et al., 2012). Mutant SOD1 is thought to aggregate in mitochondria and bind to dynein, thus disrupting axonal transport (Ligon et al., 2005; Zhang et al., 2007). This can prevent mitochondria from being recruited to areas of high energy or calcium buffering demand, and can also stop defective mitochondria from being trafficked back to the soma. Importantly, such an interaction would affect all axonal transport pathways. Recent studies in the *SOD1^G93A^* model reveal important effects on dynein-mediated transport of late endosomes. Lysosomal dysfunction occurs in a progressive manner and impairs autophagy-mediated degradation prior to symptom onset in the *SOD1^G93A^* mode. This occurs through mutant SOD1 binding with dynein, which interferes with retrograde trafficking of late endosomes, resulting in autophagic failure (Xie et al., 2015).

Mutations in *TARDBP*, the gene encoding TDP-43, are causal for ALS and like SOD1, have been shown to affect mitochondrial quality and transport. TDP-43 is a highly conserved, ubiquitously expressed DNA/RNA binding protein involved in a wide range of processes important for RNA metabolism (Baralle et al., 2013; Ling et al., 2013). In ALS, however, it was found to be ubiquitinated, phosphorylated and cleaved into insoluble C-terminal fragments that accumulate in cytoplasmic inclusions (Neumann et al., 2006; Johnson et al., 2009). TDP-43 inclusions are found in neurons and glia in both FALS and SALS, with the exception of patients and models presenting with *SOD1* mutations (Mackenzie et al., 2007; Tan et al., 2007). Transgenic mice overexpressing wild type human TDP-43 under the control of the Thy1.2 promoter, which drives expression specifically in neurons, displayed motor defects and shortened life span. Spinal motor neurons exhibited cytosolic inclusions and accumulation of fragmented mitochondria. Furthermore, mitochondria were absent from the neuromuscular junction (Shan et al., 2010). In another study, *in vivo* imaging of mitochondrial movement in mutant TDP-43 transgenic mice revealed a pre-symptomatic impairment of mitochondrial transport in motor neurons, followed by mitochondrial morphological abnormalities (Magrané et al., 2014). Mitochondrial transport defects in TDP-43 transgenic mice can be attributed to the key role that TDP-43 plays in neurofillament stability (Wang et al., 2008; Volkening et al., 2009). Together, *SOD1* and *TARDBP* mutations support a crucial requirement for mitochondrial activity and axonal transport in motor neuron function and maintenance.

## Crosstalk Between Mitochondrial and Autophagic Pathways

### Oxidative Stress and Autophagy Regulation

ROS are by-products of mitochondrial OXPHOS and include superoxide anion and peroxide, which, as a result of unpaired electrons, are unstable and reactive. ROS can readily react with virtually any macromolecule, thereby posing threat of damage to somatic or mitochondrial DNA, lipids, enzymes and other proteins. High levels of ROS lead to elevated oxidative stress within the cell, which is proposed to underlie a number of pathological states (Afanas’ev, 2005; Jang and Van Remmen, 2009). Nonetheless, oxidative stress is posited to serve a pertinent function physiologically, as increasing lines of evidence suggest a chief role in autophagy regulation (Scherz-Shouval et al., 2007; Huang et al., 2011; Scherz-Shouval and Elazar, 2011). ROS have been demonstrated to induce autophagy in starvation conditions through a mechanism requiring AMPK activation (Li et al., 2013), which drives a ROS-dependent decrease in mTOR activity. The upstream regulation of ROS-induced autophagy entails the cytoplasmic activation of ataxia telangiectasia mutated (ATM) kinase, leading to the activation of AMPK. AMPK phosphorylation of TSC2 ultimately results in inhibition of mTOR kinase (Alexander et al., 2010). Therefore, AMPK activity is required for the inhibition of mTOR, which thereby removes repression from the autophagy initiation complex to allow ULK1 phosphorylation by AMPK, triggering autophagy induction.

**Figure 3:**
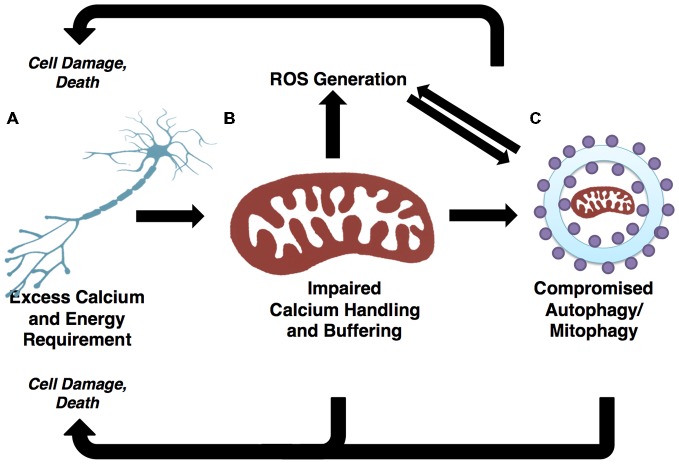
**Autophagic and mitochondrial paths to motor neuron death are convergent. (A)** Selective motor neuron degeneration is characteristic of ALS. Motor neurons’ highly polarized morphology and extensive calcium buffering and energy demands likely underlie this vulnerability. **(B)** Demands on calcium buffering and energy production are fulfilled by mitochondria. ROS are generated as a byproduct of oxidative phosphorylation (OXPHOS). The generally high metabolic requirements of neuronal cells may contribute to elevated ROS, which in turn results in cellular stress and the potential for mitochondrial dysfunction if mitophagic efficiency is not high. **(C)** Demands of neuronal mitochondria necessitate frequent turnover through mitophagy pathways. Mitochondrial-derived ROS positively regulate autophagy/mitophagy. If the autophagy/mitophagy machinery becomes impaired, ROS is not reduced and mitochondria are not recycled, thus leading to increases in ROS and cellular stress. The accumulation of ROS has potential to damage DNA, proteins and mitochondria, thereby presenting further challenge to the autophagy/mitophagy machinery. As such, the regulation of autophagy/mitophagy through mitochondrial-derived ROS and mitophagy-dependent mitochondrial quality control illustrate two pathways converging onto selective motor neuron death.

Dysfunctional and long-lived mitochondria, which are far more likely to engage in reduced-quality energy production, tend to generate excessive ROS as compared to healthy mitochondria (Chistiakov et al., 2014), thus triggering autophagic induction. This is likely a mechanism evolved to restrict damages caused by oxidative stress. Under physiological conditions, ROS are a vital component of autophagy regulation; however, when the autophagic machinery is not competent to respond, ROS levels go unchecked, presenting oxidizing damage to the cell while failing to elicit the appropriate catabolic response. It has been suggested that oxidative stress-induced dysfunction of the autophagy pathway drives the accumulation of long-lived and dysfunctional mitochondria (Luo et al., 2013). Moreover the accumulation of damaged mitochondria results in elevated oxidative stress, further challenging the autophagy-lysosome pathway in a vicious circle (Figure 3). This appears to be a prominent mechanism at play in ALS, as afflicted patients and models alike show elevated ROS and signs of unchecked oxidative stress (Weiduschat et al., 2014; Ikawa et al., 2015).

### Cell Death: The Autophagy Connection

Though oxidative stress is a signal of vital importance in promoting homeostasis and inducing autophagy, it also has a chief role in regulating cell death (Ryter et al., 2007). In fact, in models of ALS, ROS has been shown to drive motor neuron cell death (Rojas et al., 2015). Dysfunction of the autophagy-lysosome pathway under oxidative stress is a driving force for the accumulation of damaged mitochondria (Luo et al., 2013), which in turn results in heightened oxidative stress. In instances where this signal is excessive or prolonged, it may elicit a form of cell death independent from apoptosis, termed autophagic cell death (Chen et al., 2008; Li et al., 2013). One form of autophagic cell death, autosis, is characterized by independance from caspases and other constituents of pro-death pathways, as well as unique morphological features including membrane rupture, shrinkage of the nuclear membrane, electron-dense mitochondria and fragmented ER, and strengthened substrate adhesion. The autotic cell death pathway also features a unique reliance on Na^+^, K^+^-ATPases, and can be triggered pharmacologically or by starvation (Liu et al., 2013; Liu and Levine, 2015). This pathway is relevant *in vivo*, as rodent hippocampal neurons die through autosis following hypoxic-ischemic injury. That treatment with cardiac glycosides, identified in a high-throughput compound screen for autosis inhibitors, robustly rescues autotic cell death in this model suggests therapeutic potential for targeting this pathway (Liu et al., 2013). Though contributions of this pathway to motor neuron death in ALS are uncertain, it is intriguing that Na^+^/K^+^ dyshomeostasis in disease progression has been posited by computational modeling of ALS pathogenesis (Le Masson et al., 2014).

Further evidence concerning additional roles for constituents of the autophagy machinery in traditional caspase-dependent pathways implicates even broader contributions to cell death. For example, cleavage of the autophagy-related gene *Atg5* by calpain regulates apoptosis. The cleavage fragment translocates to mitochondria, where it blocks anti-apoptotic Bcl to drive cytochrome-c release and thereby promote caspase-dependent apoptosis (Yousefi et al., 2006). A similar mechanism is supported for Beclin1 in apoptotic induction (Wirawan et al., 2010). Caspase-dependent cleavage of Beclin1 yields a C-terminal fragment that localizes to mitochondria where it potentiates apoptosis, potentially through the release of pro-apoptotic factors. A role for the autophagy-associated protein ULK1 in promoting cell death, independent of autophagy, has recently emerged. Treatment with H_2_O_2_ facilitates the nuclear localization of ULK1 in an activation-dependent manner. In the nucleus, ULK1 interacts with Poly (ADP-Ribose) Polymerase 1 (PARP1), thereby increasing its activity and potentiating PARP1-induced cell death (Joshi et al., 2016). Interestingly, ULK1 is a transcriptional target of p53; its expression is upregulated in response to DNA damage, and it contributes to cell death following prolonged autophagy (Gao et al., 2011), suggesting both autophagy-dependent and—independent forms of ULK1-mediated cell death.

Motor neuron cell death in both familial and sporadic forms of ALS is attributable to necroptosis (Re et al., 2014). Necroptosis is a regulated necrotic form of cell death, independent of caspases, thereby distinguishing this pathway from apoptosis. TNF (tumor necrosis factor) induces necroptosis in a manner requiring the inactivation of caspase-8. This promotes the interaction of RIPK1 and RIPK3 (receptor interacting protein kinases), which is vital for the formation of the necrosome. The MLKL (mixed lineage kinase domain-like) pseudokinase is then recruited to execute necroptosis through an unknown mechanism (Linkermann and Green, 2014; Vanden Berghe et al., 2014). It is intriguing that some lines of evidence point to a chief role for the autophagy machinery in necroptosis. In particular, the Bcl-2 inhibitor GX15-070 promotes cells death through RIP1-dependent necroptosis, which requires autophagosome accumulation to serve as sites of necrosome assembly. Inhibition of autophagosome formation precludes this mechanism of cell death, highlighting the importance of constituents of the autophagy-lysosome pathway in necroptotic cell death. Interestingly, RIP1 inhibition does not limit GX15-070-mediated autophagosome accumulation, indicating the role of autophagy induction upstream of RIP1 activity in necroptosis (Basit et al., 2013). Altogether, the roles of autophagy in cell death are complex and incompletely understood, and contributions of these pathways to motor neuron death in ALS require further investigation. Nonetheless, that autophagic dysfunction and cell death frequently co-occur in ALS is perhaps no coincidence.

## Therapeutic Prospective

### Boosting Autophagy

That protein aggregates are characteristic of ALS and other neurodegenerative diseases suggests that the autophagy pathway may be an attractive therapeutic target for the prevention and treatment of neurodegeneration (Vidal et al., 2014). Indeed, enhancing autophagy has been shown to effectively target ALS-associated pathological aggregates for clearance to reduce toxicity (Hung et al., 2009; Wang et al., 2012; Barmada et al., 2014). The mTORC1 inhibitor rapamycin can effectively induce autophagy by eliminating mTOR-mediated inhibition of the ULK1 autophagy initiation complex (Jung et al., 2009). Rapamycin administration improved prognosis in TDP-43 mutant mice (Wang et al., 2012), but findings from trials with *SOD1* or *VCP*-associated inclusion body myopathy mutant models did not suggest a similar beneficial effect (Zhang et al., 2011; Ching et al., 2013). Interestingly, in *SOD1* mutant mice lacking mature lymphocytes, a modest survival increase was noted following rapamycin treatment (Staats et al., 2013). This finding suggests the benefit of the autophagy-promoting effect of rapamycin, as well as its detrimental effect on protective immune function. In support of this finding, preclinical trials with the autophagy-inducing agent trehalose proved successful in a *SOD1* mutant model (Castillo et al., 2013). Trehalose promotes autophagy by transcriptional upregulation of *ATG* genes through an mTOR-independent pathway.

Although direct defects in autophagy genes may not be the causal link in all cases of ALS, dysfunction of autophagy exacerbates disease phenotypes (Bruijn et al., 1998; Kitamura et al., 2014). Therefore, boosting the efficiency of autophagy could ameliorate the toxic effects of aggregates characteristic of ALS and other neurodegenerative diseases. Nonetheless, it is with some caution that we interpret these results, as there are indications that enhanced autophagy may in some instances further exacerbate axonal degeneration and disease phenotype. For example, autophagy has been implicated as an early requirement for axonal degeneration phenotypes in a number of models, including an excitotoxic neurodegeneration model (Wang et al., 2006). Moreover, the neurite retraction phenotype characteristic of the Parkinsonian *LRRK2^G2019S^* mutation implicates autophagic induction as an early event. Accordingly, RNAi-mediated knockdown of autophagy factors *LC3* or *ATG7* is sufficient to rescue neurite length (Plowey et al., 2008). In addition, it was shown using *Atg7*-deficient mice that negatively regulating autophagy is sufficient to promote cell survival of hippocampal pyramidal neurons following ischemic insult (Koike et al., 2008). These results, taken together with the expanding roles of autophagy in promoting cell death, indicate that it is the regulated balance of autophagy, not merely increased induction, that promotes neuronal health and protection.

### Enhancing Mitochondrial Health

As increasing evidence suggests a prominent role for mitochondrial dysfunction and oxidative stress in motor neuron degeneration, enhancing mitochondrial health represents a promising strategy for treating ALS and related diseases. In line with this, long-term users of the antioxidant vitamin E show a decreased risk for ALS (Wang et al., 2011), consistent with findings of elevated ROS levels (Ikawa et al., 2015) and decreased antioxidant levels (Weiduschat et al., 2014) in ALS-afflicted patients. However, most proposed antioxidant treatments for ALS are not beneficial in clinical trials (Graf et al., 2005; Orrell et al., 2008). This lack of success may be attributed to the low efficiency of targeting antioxidants to mitochondria. To circumvent this issue, the cell permeable Szeto-Schiller antioxidant peptide (SS-31) was investigated (Petri et al., 2006; Szeto, 2006). SS-31 is readily taken up by the cell and localizes to the mitochondrial inner membrane. The specific targeting of SS-31 was shown to inhibit ROS generation and protect against oxidative damage (Zhao et al., 2004, 2005). Presymptomatic treatment with SS-31 in a genetic mouse model of ALS improved disease prognosis significantly (Petri et al., 2006). It remains to be determined whether this therapeutic method offers equal potential for additional models of ALS, and how these findings will translate in human trials.

## Conclusion

Protein aggregates are chief characteristics of many neurodegenerative diseases, including ALS (Blokhuis et al., 2013). Gain-of-function properties of disease-associated mutations can directly lead to protein aggregation (Sau et al., 2007; Johnson et al., 2008; Xu et al., 2013; Wu et al., 2014). In addition, loss-of-function of *Atg* genes is sufficient to cause neurodegeneration in mice, suggesting a prominent role for autophagic clearance pathways in neurodegenerative disease (Hara et al., 2006; Komatsu et al., 2006). Consistent with this, many ALS-disease genes are linked to cellular clearance pathways (Deng et al., 2011b; Table 1), and phenotypic improvements have been achieved by enhancing autophagy in some ALS mouse models (Hung et al., 2009; Wang et al., 2012; Barmada et al., 2014). Besides impaired autophagy, defective mitochondrial function, which leads to increased oxidative stress and compromised calcium buffering, also plays a critical role in motor neuron degeneration and ALS pathogenesis. In particular, impaired autophagy and dysfunctional mitochondrial pathways may engage in crosstalk in the onset and progression of ALS. As such, therapeutic strategies targeting these two pathways and their interactions will hold great promise for alleviating disease symptoms and rescuing motor neuron degeneration in ALS and related diseases.

**Table 1:** **ALS genetics reflect a trend in impaired autophagic and mitochondrial pathways**.

Affected gene	Functional Processes/Impairment in ALS	Reference
*UBQLN2*	Ubiquitin proteasome system, autophagy	Deng et al. (2011a,b)
*SQSTM1*	Ubiquitin proteasome system, autophagy	Fecto et al. (2011)
*C9ORF72*	Repeat expansion, endosomal trafficking, autophagy	DeJesus-Hernandez et al. (2011) and Renton et al. (2011)
*VAPB*	Unfolded protein response, Ca^2+^ regulation	Nishimura et al. (2004a,b)
*OPTN*	Autophagy, mitophagy	Maruyama et al. (2010)
*VCP*	Mitophagy	Johnson et al. (2010)
*DCTN*	Axon transport	Münch et al. (2005, 2004)
*TARDBP*	DNA binding, axon transport	Kabashi et al. (2008) and Kühnlein et al. (2008)
*SOD1*	Antioxidant activity	Rosen et al. (1993)
*CHCD10*	Oxidative phosphorylation, Cristae morphology	Bannwarth et al. (2014) and Johnson et al. (2014)

*ALS-associated genes have functions relevant to degradative and mitochondrial pathways. Specifically, a number of associated genes are involved in the degradative pathways of autophagy/mitophagy and the ubiquitin proteasome system (UPS). Additionally, genes involved in mitochondrial function and quality control indicate the critical importance of mitochondrial health in neuronal maintenance and protection*.

## Author Contributions

BME, NM, and YCM conceived of, drafted, edited, and approved the corresponding review.

## Conflict of Interest Statement

The authors declare that the research was conducted in the absence of any commercial or financial relationships that could be construed as a potential conflict of interest.
